# Association between inappropriate medications and comprehensive geriatric assessment outcomes: a cross-sectional study using PRISCUS, STOPP, and Beers criteria

**DOI:** 10.1186/s12877-026-08533-0

**Published:** 2026-10-22

**Authors:** Seyma Handan Akyon, Gülşah Onur, Hande Selvi Öztorun, Kamile Sılay, Petra A. Thürmann

**Affiliations:** 1 Family Medicine Department, Home Health Care Unit, Sincan Training and Research Hospital, Ankara, Türkiye; 2 Muş Bulanık District Health Directorate, Muş, Türkiye; 3 Ankara Yildirim Beyazit University, Faculty of Medicine, Department of Geriatrics, Ankara Bilkent City Hospital, Department of Geriatrics, Ankara, Türkiye; 4 Chair of Clinical Pharmacology, Faculty of Health, Department of Medicine, Helios University Hospital Wuppertal, University Witten/Herdecke, Witten, Germany

**Keywords:** Geriatric assessment, Potentially inappropriate medication list, Primary health care, Drug-related side effects and adverse reactions, Polypharmacy

## Abstract

**Aim:**

Different criteria were developed to identify drugs with a negative benefit/risk ratio in older adults, so-called potentially inappropriate medication (PIM). We investigated the correlation between the number of PIMs identified using PRISCUS 2.0, STOPP version 3, and Beers criteria and the functional and nutritional status of Turkish geriatric patients, as assessed by comprehensive geriatric assessment (CGA) tools.

**Methods:**

This retrospective cross-sectional analysis was conducted in geriatric outpatients over the past 10 months. Data were collected on age, clinical conditions, medication, and CGA test results. To identify independent factors associated with PIM use and to adjust for potential confounding, logistic regression analyses were performed. PIM use was analysed as a binary outcome (no PIM versus at least one PIM), and no imputation was performed. Two logistic regression models were fitted in 362 patients with complete data, without (Model 1) and with (Model 2) the number of drugs.

**Results:**

Among 397 participants, 58.7% (*n* = 233) of patients received at least one PIM when combining all three criteria (PIM-ALL). Multivariable analyses showed that the number of medications and functional status (Katz Activities of Daily Living) were most strongly associated with PIM use. In the fully adjusted model (Model 2, *n* = 362), each additional medication was associated with 55% higher odds of PIM use (OR 1.55, 95% CI 1.40–1.73), while a higher Katz ADL score was associated with lower odds (OR 0.73, 95% CI 0.60–0.87).

**Conclusion:**

There is relevant variability among PIM tools and in their association with CGA test results. Advanced, systematic, and easily implementable methodologies are required to address these shortcomings.

**Clinical trial registration:**

This study does not involve a clinical trial and thus was not registered.

**Supplementary Information:**

The online version contains supplementary material available at https://doi.org/10.1186/s12877-026-08533-0.

## Introduction

The aging population has brought significant challenges to healthcare systems, particularly in managing multimorbidity and polypharmacy, usually described as the use of more than five medications [1]. Potentially inappropriate medications (PIMs), defined as medications where the potential risk outweighs the potential benefit, are of critical concern in this context, as they increase the risk of adverse drug reactions (ADRs), morbidity, mortality, and economic burden [2–4]. Older people are more vulnerable to ADRs due to physiological changes and experience a high prevalence of PIMs [5]. Studies show up to 61% of hospitalized older adults experience preventable ADRs, highlighting the need to identify and address PIMs, particularly given the existence of safer alternatives for similar conditions [6]. Given the substantial impact of PIMs on health outcomes and healthcare resources, developing and applying effective criteria is essential for improving prescribing practices and ensuring medication safety in older adults.

Several criteria exist to identify PIMs in older adults, notably the American Geriatrics Society (AGS) Beers Criteria [7], the screening tool of older persons’ potentially inappropriate prescriptions (STOPP) criteria [8], and the German PRISCUS list [9]. Beers and STOPP address disease states and drug interactions, focusing on specific medications and drug classes. PRISCUS offers a diagnosis-independent list, identifying more PIMs and suggesting alternatives. All criteria used the Delphi consensus method to gather expert opinions systematically [10, 11].

While PIM avoidance is a key strategy for reducing adverse events in older adults, the association between PIM use and ADRs like functional decline, confusion, falls, and mortality remains unclear, prompting increasing systematic review efforts [12]. Among older adults with dementia across eight European countries a high prevalence of PIM use (60%) was reported, which was associated with increased risks of fall-related injuries and hospitalizations, especially in individuals aged 80 years and older or those with higher comorbidities [13]. For the Beers and STOPP criteria, multiple studies showed an association between PIM use and adverse outcomes such as avoidable hospitalizations and functional impairments. However, evidence for an association between the use of drugs from the updated PRISCUS 2.0 list and ADRs is limited [14]. In addition, due to international differences in drug markets, some tools may have limited use in other countries, as shown by Fialova et al. [15, 16].

This study aims to evaluate the association between PIMs identified by the 2019 AGS Beers, STOPP version 3(v3), and PRISCUS 2.0 criteria and various geriatric characteristics, including functional and nutritional status, using comprehensive geriatric assessment (CGA) tools. Furthermore, we compared the associations of these three criteria with adverse outcomes, including hospitalization and mortality.

## Methods

### Study participants

This retrospective, monocentric cross-sectional study included all patients aged 65 years and older who applied to the Geriatrics Outpatient Clinic at Ankara Bilkent City Hospital over a 10-month period (March 15, 2023, to January 15, 2024). Data on age, clinical conditions (including diseases, history of delirium, fractures, falls, glomerular filtration rate, hospitalization, mortality, and ejection fraction rate), and medication use (duration and doses) were retrieved from patients’ electronic health records (EHRs). CGA results were recorded using standardized tools that encompassed cognitive, functional, and psychosocial parameters. Ethics committee approval was obtained before the study. All data were extracted and analyzed anonymously in accordance with national data protection regulations.

### Ethics approval statement

This study was conducted in accordance with ethical principles and approved by the Ankara Bilkent City Hospital Clinical Research Ethics Committee on 6 December 2023, with document number E2-23-5844.

### Comprehensive geriatric assessment tools

CGA included the following validated tools: Mini Nutritional Assessment Short Form (MNA-SF) [17], Yesavage’s Geriatric Depression Scale Short Form (GDS-15) [18], Katz Activities of Daily Living (ADL) scale [19], and Lawton Brody Instrumental Activities of Daily Living (IADL) scale [20].

### PIM criteria

#### 2019 AGS Beers criteria

The Beers Criteria, introduced in 1991 by the AGS, are widely used for identifying PIMs. The 2019 version includes five sections covering medications to avoid, drug interactions, renal dose adjustments, and drugs that should be used with caution [7].

#### The STOPP criteria v3

The STOPP criteria, developed in Ireland in 2006, use a systems-based approach to identify PIMs, drug duplications, and drug-disease interactions. STOPP v3, published in 2023, was utilized in this study, encompassing 133 updated items reflecting current clinical guidelines and prescribing practices [8].

#### PRISCUS 2.0 list

The PRISCUS 1.0 List, introduced in Germany in 2010 and updated as PRISCUS 2.0 in 2023, includes 177 drugs’ PIMs. It provides detailed recommendations, including treatment alternatives and guidance on monitoring. PRISCUS 2.0 expanded significantly on the original, incorporating new drug categories [9].

### PIM classification

Using the PRISCUS list, Beers criteria, and STOPP criteria, PIMs were classified into three categories: PIM-A, PIM-C, and PIM-ALL **(**Table 1**).** Operationalizing all criteria from STOPP Version 3 and Beers 2019 presented challenges, particularly for parameters not fully documented in the EHR. For instance, the STOPP criterion regarding statin use for primary cardiovascular prevention in individuals aged 85 years or older with established frailty and limited life expectancy required assumptions, as frailty and life expectancy were not explicitly recorded in the EHR. Similarly, the Beers 2019 criterion regarding aspirin use for primary prevention requires clear documentation of the indication. As this information was not always available in the electronic health records (EHRs), medications were counted as potentially inappropriate only when sufficient information was available to make a clear decision. For this purpose, prescription duration, dosage, and continuity of use were verified using EHR data. Treatment duration was determined by reviewing the EHRs over an extended time period, including previous prescriptions, prescription issue dates, pharmacy dispensing records, and documented dosing information. Repeated prescriptions and continuous dispensing were interpreted as evidence of long-term use. Medications were classified as PIMs only when these data clearly met the Beers, STOPP, or PRISCUS criteria. If the indication or duration of use could not be clearly determined, the medication was not counted as a PIM. The full operational definition of PIM-A, PIM-C, and PIM-ALL, including the rule used to allocate each individual criterion item to one of these groups and worked examples for each source list, is provided in Supplementary File 1 (Tables S1 to S5). Condition-dependent (PIM-C) criteria, including those relating to dementia, were applied on the basis of clinical diagnoses documented in the EHR; cognitive test scores or score-based cut-offs were not used for PIM classification.

**Table 1:** PIM classification and descriptions

PIM Classification	Description
PIM-A*	Medications considered inappropriate due to **age**, dosage, or prescription duration, as outlined in the PRISCUS list, Beers, and STOPP criteria.
PIM-C**	Medications were classified as inappropriate based on the patient’s clinical **conditions**, co-existing diseases, prescription details, laboratory findings, and documented clinical events, as indicated in Beers and STOPP criteria.
**PIM-ALL**	A comprehensive classification combining PIM-A and PIM-C, based on the PRISCUS list, Beers criteria, and STOPP, provides a **holistic** view of PIMs.

*Medications were classified as PIM-C if their inappropriateness was determined based on clinical conditions, co-existing diseases, or specific laboratory findings alongside their dosage or prescription duration, as outlined in Beers and STOPP criteria. In contrast, medications considered inappropriate solely due to age, dosage, or prescription duration, without requiring a clinical condition or co-morbidity for classification, were categorized under PIM-A, following the PRISCUS list, Beers, and STOPP criteria

**Drug-drug interactions, and laboratory findings such as glomerular filtration rate (GFR) and ejection fraction were analyzed based on Beers and STOPP criteria and categorized under PIM-C. Additionally, documented clinical events, including history of delirium, fractures, falls, and hospitalizations, were incorporated into the evaluation. For example, medications contraindicated in diseases such as dementia (e.g., antipsychotics, anticholinergics), urinary incontinence (e.g., alpha-blockers), delirium (e.g., benzodiazepines, antipsychotics), and heart failure (e.g., NSAIDs, digoxin) were identified from clinical diagnoses documented in the EHR and classified accordingly. Criteria based solely on dose or duration of use (e.g., PPI > 8 weeks, NSAID > 3 months) were categorized under PIM-A. This comprehensive approach ensured that patient-specific factors, including both clinical conditions and events, were included in the assessment of PIMs

### Statistical analyses

The Statistical Package for the Social Sciences (SPSS) for Windows, version 26.0 (IBM SPSS Inc., Chicago, IL), was used for the descriptive analyses. The logistic regression analyses were conducted using R (Version 4.3.3; R Core Team, 2024) within RStudio (Version 2026.04.0; Posit Software, 2026). We examined the conformity of variables to a normal distribution using both visual (histograms and probability graphs) and analytical (Kolmogorov-Smirnov/Shapiro-Wilk tests) methods. We presented the descriptive analysis results as means and standard deviations for variables with a normal distribution, and as medians and minimum-maximum ranges for variables without a normal distribution. We gave the frequency of categorical variables as a percentage (%). Covariates for the multivariable models were pre-specified on clinical grounds rather than selected by their bivariate p values. Age and sex were included as standard demographic confounders, the number of drugs was included as the measure of medication burden, and Katz ADL, MNA and recurrent falls were included because functional dependency, nutritional status and falls are well-documented correlates of PIMs in older adults. Medication burden was entered in one form only, as a continuous drug count, and the polypharmacy and hyperpharmacy categories were not entered alongside it because they are deterministic thresholds of the same variable. No stepwise or automated selection procedure was applied. Two model families were fitted: Model 1 contained age, sex, Katz ADL, MNA and recurrent falls, and Model 2 added the number of drugs. Both models were estimated in the 362 patients with complete data for all covariates. Complete-case analysis was used throughout and missing values were not imputed.

## Results

### Descriptive analyses

The study included 397 participants, with a median age of 77 years (range, 65–105 years). Most participants were female (59.4%, *n* = 236). The median number of drugs used by participants was 5 (range 0–20), and we found that 56.9% (*n* = 226) used polypharmacy. The median number of chronic diseases per patient was 3 (range 0–9), and 89.1% (*n* = 354) had two or more chronic conditions.

### Clinical and functional status

A history of delirium was reported in 3% (*n* = 12) of the participants, while 29.2% (*n* = 116) had a history of falls. The median number of falls in the last year was 0 (range 0–8), and 5.5% (*n* = 22) of participants experienced recurrent falls. The median number of hospitalizations in the past year was 0 (range 0–6). Mortality rates within 6 and 12 months were 8.1% (*n* = 32) and 10.3% (*n* = 41), respectively.

### Comprehensive geriatric assessment

This study assessed CGA scores across different domains; however, not all 397 patients had data available for every scale. The Katz ADL had data for 374 patients (94.2%), the Lawton-Brody IADL score for 371 (93.5%), the MNA for 362 (91.2%), and the GDS-15 for 281 (70.8%).

### PIM classification

Table 2 presents the distribution of PIM according to the Beers, PRISCUS, and STOPP criteria, highlighting the median values, the percentage of patients using at least one PIM, the total number of PIMs identified, and the most frequently prescribed medications for each criterion. The number of PIM-ALL items per patient ranged from 0 to 7 (0: 41.3%, 1: 25.9%, 2: 17.6%, 3 or more: 15.1%; Supplementary File 1, Table S5).

**Table 2:** Distribution of PIM according to Beers, PRISCUS, and STOPP criteria with most frequently prescribed medications

Criteria	Median (min-max)	Rate of patients using at least one PIM,% (*n* = 397)	Total number of PIM	Five most frequent PIMs (PIM-A and PIM-C combined)*,, % (*n* = 397)
Beers-PIM A	0(0–3)	32.0% (*n* = 127)	151	
Beers-PIM C	0(0–6)	26.2% (*n* = 104)	158	
Beers-PIM A + C	0(0–6)	46.1% (*n* = 183)	296	PPI (23.2%, *n* = 92) quetiapine (9.8%, *n* = 39 ) gliclazide (4.5%, *n* = 18) doxazosin (3%, *n* = 12) insulin (2.8%, *n* = 11)
PRISCUS-PIM A	0(0–5)	49.9% (*n* = 198)	328	PPI (23.4%, *n* = 93) quetiapine (8.6%, *n* = 34) gliclazide (5.5%, *n* = 22) NSAID (5%, *n* = 20) trimetazidine (3.8%, *n* = 15)
STOPP-PIM A	0(0–3)	30.7% (*n* = 122)	147	
STOPP-PIM C	0(0–4)	22.7% (*n* = 90)	130	
STOPP-PIM A + C	0(0–5)	43.3% (*n* = 172)	274	PPI (23.2%, *n* = 92) quetiapine (6%, *n* = 24) gliclazide (5.5%, *n* = 22) furosemide (5.0%, *n* = 20) ASA (3%, *n* = 12)
PIM-ALL	1(0–7)	58.7% (*n* = 233)	480	PPI (23.4%, *n* = 93) quetiapine (9.8%, *n* = 39) gliclazide (5.5%, *n* = 22) NSAID (5%, *n* = 20) furosemide (5.0%, *n* = 20)

**Abbreviations**: Proton pump Inhibitor (PPI), Nonsteroidal anti-inflammatory drug (NSAID), Acetylsalicylic acid (ASA)

*The classification of PPIs, NSAIDs, and other medications as PIMs was determined based on the prescribed dose and duration, aligning with the specific thresholds outlined in Beers, STOPP, and PRISCUS criteria. For example, PPIs prescribed for > 8 weeks without a clear indication, NSAIDs used for > 3 months, benzodiazepines used for > 4 weeks, or acetylsalicylic acid at doses > 100 mg/day were classified as inappropriate

### Regression analyses

PIM-ALL was analysed as a binary outcome (no PIM versus at least one PIM) using the pre-specified covariate set described in the Methods; the full results of both models are given in Table 3. In Model 1 (*n* = 362), a higher Katz ADL score was associated with lower odds of PIM use and recurrent falls with higher odds. Adding the number of drugs in Model 2 (*n* = 362) identified medication burden as the strongest correlate of PIM use, with each additional medication increasing the odds by 55% (OR = 1.547, 95% CI 1.400-1.727; *p* < 0.001), while the Katz ADL association persisted and recurrent falls were no longer associated with PIM use; Nagelkerke’s R² rose from 0.160 to 0.459. MNA was not associated with PIM use in either model.

**Table 3:** Multivariable logistic regression models for the presence of at least one PIM (PIM-ALL, 0 vs. ≥ 1)

Variable	Model 1 Odds Ratio (95% CI)	*P*-value	Model 2 Odds Ratio (95% CI)	*P*-value
N (complete cases)	362	–	362	–
Age	1.013 (0.980–1.047)	0.456	0.999 (0.963–1.038)	0.979
Sex (male vs. female)	0.865 (0.545–1.373)	0.537	0.898 (0.520–1.553)	0.699
Number of drugs (continuous)	–	–	**1.547 (1.400–1.727)**	**< 0.001**
Katz ADL (per-point)	**0.692 (0.584–0.809)**	**< 0.001**	**0.725 (0.597–0.870)**	**0.001**
MNA (metric)	1.015 (0.925–1.114)	0.749	1.029 (0.924–1.146)	0.608
Recurrent falls (yes vs. no)	**4.424 (1.439–19.328)**	**0.020**	1.752 (0.496–8.275)	0.420
Nagelkerke’s R²	0.160	––	0.459	––

Model 1: age, sex, Katz ADL, MNA and recurrent falls

Model 2: Model 1 plus the number of drugs (continuous). Both models were estimated in patients with complete data (*n* = 362). Bold indicates *p* < 0.05

## Discussion

### PIM criteria

The results showed that PIM-ALL identified the highest prevalence of PIM use, followed by PRISCUS-PIM-A, Beers-PIM-A + C, and STOPP-PIM-A + C, with proton pump inhibitors(PPIs) being the most commonly prescribed PIM across all criteria. These findings align with previous studies comparing the STOPP and Beers criteria [21, 22]. The updated PRISCUS 2.0 list encompasses a broader range of drug classes, featuring detailed monitoring and alternative treatment recommendations. This revision aligns more closely with other explicit PIM criteria, as reflected in our findings, in which the prevalence of PIMs identified by PRISCUS 2.0 approached that of Beers and STOPP [11]. The comprehensive approach of PIM-ALL, integrating all three criteria, underscores the importance of a multi-faceted strategy for identifying PIMs. Its higher prevalence rate highlights the necessity of broader frameworks to capture the complexity of inappropriate prescribing and to provide a more inclusive assessment of medication safety in older adults. Comparative work across cohorts has repeatedly shown that agreement between explicit criteria is moderate at best and that their predictive validity for hard clinical outcomes varies by version and by setting [22, 23]. This is one reason we report Beers, STOPP, and PRISCUS separately as well as combined in PIM-ALL, rather than treating them as interchangeable measures of the same underlying exposure. A review from the same group traced the move from the Beers criteria to STOPP/START and noted that the Beers list is not easily applied in European settings, since several of the listed drugs are not marketed there [24].

The direct association between PIM use and specific adverse outcomes remains complex [25]. In the regression analyses, overall medication burden and functional status showed the strongest associations with PIM use; age and sex were not associated with PIM use, and the association with recurrent falls did not persist after adjustment for the number of drugs. We did not observe significant associations between PIMs and hospitalization, delirium, or mortality; given the small number of these events, these findings are more consistent with limited statistical power than with an absence of association. The literature supports the association between polypharmacy and PIMs, with increasing medication use elevating PIM risk due to factors like prescribing errors and fragmented care [26].

A multi-center cohort study, based on the PRISCUS 1.0 list, found that PIMs and drug-drug interactions increase the risk of hospitalizations and falls but not mortality in older adults [27]. In a study examining medication-related falls in older adults hospitalized with hip fractures, 41% of falls were thought to be medication-related. Notably, 90% of these cases included at least one PIM identified by the STOPP criteria, underscoring the critical role of medication reviews in reducing fall risk among older adults [28]. Similarly, a study using the Beers criteria in a sample of 387 older outpatients found that 55% were at high risk of falls, with a significant association observed between polypharmacy, PIMs, and fall risk [29]. In our study, patients with recurrent falls tended to have higher odds of PIM use, but this association lost statistical significance after adjustment for medication load. This weakening may indicate that the association between recurrent falls and PIM prescribing is largely accounted for by overall medication burden and that our study had limited power to detect an independent association.

Our study did not find a significant link between PIM use and hospitalization across all criteria; however, findings from the PRISCUS 1.0 list and a study suggest that PIM use may increase the risk of all-cause hospitalization compared to alternatives within 180 days post-dispensing [14]. Additionally, evidence suggests that PIMs identified by the STOPP criteria are significantly associated with avoidable ADRs that cause or contribute to urgent hospitalizations [30]. However, the evidence regarding hospitalization and other healthcare outcomes associated with the Beers criteria remains inconsistent, particularly across different healthcare settings, highlighting the need for improved predictive validity [31]. A study using the Beers criteria reported increased hospitalization and mortality risks in nursing home residents receiving PIMs [32]. The clinical characteristics of the study population may have influenced the detection of these associations. A simpler explanation comes from prospective data in a similar setting. Fabbietti et al. followed 647 older patients for three months after discharge from acute care hospitals. Polypharmacy, defined there as more than eight medications, was associated with readmission, while Beers violations, STOPP violations, and the two combined were not. Polypharmacy remained associated with readmission whichever criterion was included in the model, and the authors concluded that this association was largely independent of PIM use [33]. Our null result for hospitalization may reflect a similar pattern, in which the number of drugs rather than the criteria applied to them carries the association with admission. Further reasons for the relatively weak association between hospitalization and mortality may include sample size, the sensitivity of the criteria, or heterogeneity in the analyzed population. The literature contains conflicting findings regarding the association between PIMs and these outcomes.

The application of PIM lists in clinical practice is challenging due to the extensive information provided and the difficulty of aligning with local drug markets and prescribing habits [34]. Another significant challenge, particularly in primary care, is the limited awareness of PIM lists as valuable tools for evidence-based risk-benefit analyses, which hinders their widespread adoption in routine practice [35]. According to a study conducted in Germany, although the use of PIMs has been declining, regional differences indicate considerable room for improvement, highlighting the necessity of regular updates to reflect growing evidence and provide prescribers with up-to-date guidance [36]. These practical barriers also limit data availability on PIM prescribing, complicating efforts to enhance medication safety for older adults. While these tools provide quick, standardized guidance for decision-making, they offer general recommendations and alternatives rather than individualized patient-specific advice [11].

### Comprehensive geriatric assessment

In the fully adjusted model (Model 2, *n* = 362), each additional medication was associated with 55% higher odds of PIM use, while a higher Katz ADL score was associated with lower odds. At the same time, nutritional status (MNA) showed no significant association.

#### Functional status

In our multivariable logistic regression, functional independence showed a concurrent inverse association with PIM use: each one-point increase in Katz ADL score was associated with approximately 27% lower odds of having at least one PIM. Because exposure and outcome were recorded at the same visit, the reverse direction is equally plausible, in that patients with poorer functional status may receive more medications and therefore accumulate more PIMs. These findings align with a systematic review using STOPP and Beers criteria, which demonstrated significant associations between PIM and impaired physical function, including ADL and IADL scores, slower walking speed, reduced grip strength, and prolonged recovery times, further validating our results [14]. In our regression models, higher Katz ADL scores were independently associated with lower odds of PIM use, indicating that more functionally independent patients were less likely to receive PIMs. Some studies question PIMs’ impact on functional status, with a large post-hoc analysis finding no significant association of Beers-identified PIMs with Barthel scores or walking ability in geriatric patients [37]. This suggests that the relationship between PIM use and functional outcomes may vary depending on the population, methodology, and assessment criteria. Our observation that overall medication burden showed the strongest association with PIM exposure can be read alongside a multicentre Italian cohort of 733 older patients followed after discharge from acute care hospitals. In that study, hyperpolypharmacy and Beers violations were each associated with functional decline after adjustment, whereas STOPP violations were not [23]. This suggests that drug burden and the explicit criteria may carry partly separate information, and that the criteria differ in how they relate to function. That cohort was followed over time, while our design is cross-sectional, so these results do not indicate the direction of our Katz ADL association.

#### Cognitive function and nutrition

After adjustment for age, sex, functional status, recurrent falls, and medication load, nutritional status (MNA) was not independently associated with PIM use, and the adjusted odds ratios were close to unity. Cognitive performance could not be examined in this analysis (see Limitations). Previous studies reported associations between PIM exposure and reduced cognitive performance [38], worsened cognitive outcomes among cognitively impaired nursing home residents [39], lower MNA scores with higher PIM use and adverse drug reaction risk [40]. A German longitudinal study found no consistent association between cognitive decline and PIMs, although its cross-sectional analyses showed higher odds of PIM use among cognitively impaired patients [41]. Given the low prevalence of severe malnutrition in this cohort, and differences in case-mix and prescribing culture, this null finding is more consistent with limited statistical power than with an absence of association, and we do not interpret it further. The established links between malnutrition, polypharmacy, and adverse outcomes [42, 43] nonetheless support integrating nutritional assessment into medication review.

### Limitations

This cross-sectional design precludes any causal inferences and does not allow assessment of longitudinal changes or long-term outcomes. Some geriatric assessment and medication data may be incomplete or in part based on patient self-report, and missing or inconsistent electronic health records entries for key parameters (e.g. frailty, life expectancy, detailed fall or syncope history) limited the full application of several STOPP and Beers criteria. Cognitive function was assessed in routine care but was not included in the analyses because the licensing requirements for the cognitive test instrument remained unresolved, so cognitive impairment may act as an unmeasured confounder. Clinical diagnoses of dementia documented in the EHR were still used for PIM-C classification. Missing CGA data were substantial for the GDS-15, and no advanced missing data strategy such as multiple imputation was applied. The multivariable models are therefore complete-case analyses. Depressive symptoms were not included in the models because GDS-15 data were missing for 29% of patients. Because exposure and outcomes were recorded at the same visit, the reported associations are concurrent and cannot establish direction. Because clinical conditions relevant to condition-dependent (PIM-C) criteria were not captured as a discrete, structured variable in our database, cases in which such information was not documented in the medical records could not be distinguished from cases in which the condition was genuinely absent; consistent with the STOPP/Beers methodology, undocumented conditions were treated as ‘criterion not met,’ which may have led to an underestimation of PIM-C prevalence and represents a potential source of misclassification; the maximum extent of this under-ascertainment is shown per item in Supplementary File 1(Table S4). In addition, the single-centre setting and the inherent heterogeneity of health status and medication use in older adults restrict the generalizability of our findings. Future studies should use longitudinal designs and more CGA to identify risk factors and markers for PIM use, ideally combining detailed PIM classification with multivariable regression models.

## Conclusion

In conclusion, total medication count and reduced functional status (Katz ADL) were consistently associated with PIM use, whereas nutritional status (MNA) showed no significant association. These associations are concurrent and do not indicate that PIMs cause functional decline. While comprehensive tools like PIM-ALL capture the overall burden of PIMs, condition-specific tools like STOPP and Beers provide deeper insights in tailored clinical contexts. With its straightforward approach, the PRISCUS criteria remain a practical option for time-sensitive settings. Enhancing the identification and management of PIMs in clinical practice requires advanced, systematic, and easily implementable methodologies that overcome the limitations of existing criteria. Such approaches can pave the way for more personalized and effective medication management, ultimately leading to improved outcomes for older adults.

## Supplementary information

Below is the link to the electronic supplementary material.

Supplementary Material 1

## Data Availability

The data supporting this study’s findings are not publicly available due to concerns regarding safety and confidentiality.
