# Supplementary material for: Association between inappropriate medications and comprehensive geriatric assessment outcomes: a cross-sectional study using PRISCUS, STOPP, and Beers criteria
Source: BMC Geriatr. 2026 Oct 22;26:1351. doi: 10.1186/s12877-026-08533-0 (PMC13700195; doi:10.1186/s12877-026-08533-0)
Supplement: Supplementary file 1 [file 12877_2026_8533_MOESM1_ESM.docx]

# **Supplementary File 1. Operational Definitions and Reproducible Classification Criteria for PIM-A, PIM-C, and PIM-ALL Categories**

This supplementary file was prepared in response to the reviewer comment that the PIM-A / PIM-C / PIM-ALL grouping lacked transparency. It provides an explicit, reproducible operational definition of how individual medications were allocated to each category, the specific criterion that triggered each classification (age, fixed dose, duration of use, clinical condition, laboratory parameter, documented clinical event, or drug-drug interaction), and the corresponding frequency observed in the study cohort (N = 397 patients). Medication names are reported by International Nonproprietary Name (INN). Table S4 quantifies, for the most consequential condition-dependent items, the number of patients in whom the item could not be adjudicated, and Table S5 gives the distribution of the number of PIM-ALL items per patient. Abbreviations: ASA, acetylsalicylic acid; NSAID, non-steroidal anti-inflammatory drug; PPI, proton pump inhibitor; GFR, glomerular filtration rate; EF, ejection fraction; BPH, benign prostatic hyperplasia.

## Operational Definitions

Table S1 summarizes the operational definition of each PIM category and the specific classification rule applied in this study.

**Table S1. Operational definitions of PIM-A, PIM-C, and PIM-ALL and the classification rule applied in this study.**

| PIM Category | Definition | Classification rule applied in this study |
| --- | --- | --- |
| PIM-A | Medications considered potentially inappropriate on the basis of age, fixed maximum dose, or maximum recommended duration of use (PRISCUS 2.0, Beers 2019, STOPP v3), independent of the patient’s clinical condition. | Coded as PIM-A whenever the source list flagged the item solely because the patient was above the specified age threshold, the dose exceeded a fixed maximum, or the duration of use exceeded a fixed maximum, with no requirement for a co-existing disease, laboratory abnormality, or clinical event. |
| PIM-C | Medications considered potentially inappropriate on the basis of the patient’s clinical condition, comorbidity, prescription details, laboratory findings, or a documented clinical event (Beers 2019, STOPP v3). | Coded as PIM-C whenever the source list required, in addition to the medication itself, a specific comorbidity/disease documented as a clinical diagnosis in the EHR (e.g., dementia), an abnormal laboratory parameter (e.g., eGFR, potassium, ejection fraction), a documented clinical event (fall, fracture, delirium, hospitalization), or an interacting co-medication (drug-drug interaction). |
| PIM-ALL | A comprehensive classification combining PIM-A and PIM-C, based on PRISCUS 2.0, Beers 2019, and STOPP v3, providing a holistic view of PIMs. | Calculated as the de-duplicated union of all PIM-A and PIM-C items identified for a given patient across the three reference lists (i.e., a medication flagged by more than one list is counted only once). |

## Reproducible Classification Algorithm

Step 1. For every medication in a patient’s regimen, check whether it appears on the PRISCUS 2.0, Beers 2019, and/or STOPP v3 reference lists used in this study.

Step 2. For each match, identify the specific sub-criterion invoked by that list: an age threshold, a fixed maximum dose, or a maximum recommended duration of use, versus a specific comorbidity/disease, a laboratory parameter (eGFR, potassium, ejection fraction), a documented clinical event (fall, fracture, delirium, hospitalization), or a drug-drug interaction.

Step 3. If the sub-criterion is age, dose, or duration only (independent of clinical context), code the item as PIM-A for that list. If the sub-criterion additionally requires a clinical condition, laboratory abnormality, documented event, or an interacting co-medication, code the item as PIM-C for that list.

Step 4. Repeat Steps 1-3 independently for each of the three reference lists (PRISCUS 2.0, Beers 2019, STOPP v3), yielding list-specific PIM-A and PIM-C counts.

Step 5. PIM-ALL is calculated as the de-duplicated union of all PIM-A and PIM-C items identified across the three lists for that patient; a medication flagged by more than one list is counted once.

## List-Specific Coding Detail (N = 397 patients)

For each of the three reference lists, the algorithm above was applied item-by-item to the full cohort. The resulting PIM-A item list (drug, triggering sub-criterion, and number of patients in whom it was flagged) and the PIM-C items (drug paired with the qualifying clinical condition, laboratory finding, or interacting co-medication, and the corresponding number of patients) are reported below. In Tables S2 and S3, n refers to items (drug-condition pairs): a drug-drug interaction counts as one item, and a drug flagged under two different triggers is listed under each trigger. In Table 2 of the main text, counts refer to distinct medications per patient, so a two-drug interaction contributes two medications and a drug flagged under two triggers contributes one. The two totals therefore differ slightly (Beers 2019 PIM-C: 160 items vs 158 medications; STOPP v3 PIM-C: 122 items vs 130 medications). PIM-A totals are identical in both (Beers 2019: 151; PRISCUS 2.0: 328; STOPP v3: 147).

### Beers Criteria 2019

PIM-A (age/dose/duration-dependent), n = 18 unique items:

PPI/proton pump inhibitor (n=92, duration); gliclazide (n=18, age); short-/intermediate-acting insulin (n=11, age); doxazosin (n=10, age); amitriptyline (n=6, age); glimepiride (n=2, age); olanzapine (n=1, age); sulpiride (n=1, age); clonazepam (n=1, age); paroxetine (n=1, age); atropine (n=1, age); amiodarone (n=1, age); metoclopramide (n=1, age); doxylamine (n=1, age); chlorzoxazone (n=1, age); lorazepam (n=1, age); hydroxyzine (n=1, age); alprazolam (n=1, age).

PIM-C (clinical condition/laboratory/event- or interaction-dependent), grouped by triggering domain (Table S2):

**Table S2. Beers 2019 PIM-C items grouped by triggering clinical domain (N = 397).**

| Clinical domain / trigger | Total n | All items (drug — condition, n) |
| --- | --- | --- |
| Dementia | 54 | quetiapine (n=36); olanzapine (n=6); trospium (n=3); fesoterodine (n=2); haloperidol (n=2); risperidone (n=2); aripiprazole (n=1); propiverine (n=1); tolterodine (n=1) |
| History of falls | 44 | escitalopram (n=10); citalopram (n=6); quetiapine (n=6); sertraline (n=6); duloxetine (n=4); olanzapine (n=2); amitriptyline (n=1); carbamazepine (n=1); fentanyl (n=1); haloperidol (n=1); paroxetine (n=1); pregabalin (n=1); trifluoperazine (n=1); tramadol (n=1); opioid, unspecified (n=1); SSRI, unspecified (n=1) |
| Drug-drug interaction | 20 | NSAID + clopidogrel (n=3); NSAID + ASA (n=2); NSAID + NSAID + CKD (n=1); NSAID + rivaroxaban (n=1); quetiapine + olanzapine (n=1); quetiapine + haloperidol (n=1); quetiapine + citalopram + olanzapine (n=1); quetiapine + aripiprazole + sertraline + trazodone (n=1); clonazepam + quetiapine + sertraline (n=1); gabapentin + quetiapine + tramadol (n=1); sertraline + sulpiride + gabapentin (n=1); sertraline + trazodone + pregabalin (n=1); pregabalin + tramadol + duloxetine (n=1); pregabalin + tramadol + quetiapine + escitalopram + fentanyl + olanzapine (n=1); amitriptyline + duloxetine + sitilizan (n=1); aripiprazole + alprazolam + tramadol (n=1); levetiracetam + venlafaxine + primidone (n=1) |
| History of fracture | 11 | quetiapine (n=2); alprazolam (n=1); amitriptyline (n=1); aripiprazole (n=1); codeine (n=1); duloxetine (n=1); escitalopram (n=1); gabapentin (n=1); sertraline (n=1); tramadol (n=1) |
| Urinary incontinence / BPH | 9 | doxazosin (n=4); alverine (n=1); atropine (n=1); fesoterodine (n=1); tolterodine (n=1); trospium (n=1) |
| Low GFR (renal impairment) | 7 | levetiracetam (n=3); pregabalin (n=2); famotidine (n=1); apixaban (n=1) |
| Heart failure / low EF | 5 | digoxin in HFpEF (n=3); diltiazem (n=1); NSAID (n=1) |
| History of delirium | 5 | quetiapine (n=3); lorazepam (n=1); olanzapine (n=1) |
| Hypertension | 2 | doxazosin (n=2) |
| Parkinson’s disease | 1 | aripiprazole (n=1) |
| Atrial fibrillation | 1 | amiodarone (n=1) |
| Other clinical condition | 1 | oxybutynin + propiverine (n=1) |

### PRISCUS 2.0

The PRISCUS 2.0 list is structured predominantly as an explicit, age-based inventory of medications to avoid or use with caution in patients aged 65 years or older; unlike Beers 2019 and STOPP v3, it contains very few criteria that are conditional on comorbidity or laboratory findings. Consistent with this structure, no PIM-C item was identified under PRISCUS 2.0 in the present cohort (all 328 PRISCUS-flagged medication entries were coded PIM-A).

PIM-A (age/dose/duration-dependent), n = 52 unique items:

PPI (n=93; age n=3, dose n=10, duration n=80); quetiapine (n=34; dose n=2, duration n=32); gliclazide (n=22, age); NSAID (n=20, age); trimetazidine (n=15, age); doxazosin (n=12, age); Ginkgo biloba (n=9, age); olanzapine (n=8, age); pramipexole (n=9, age); digoxin (n=8, age); betahistine (n=7, age); piracetam (n=7, age); trospium (n=7, age); tramadol (n=7, age); rasagiline (n=5, age); propiverine (n=4, age); cilostazol (n=5, age); solifenacin (n=3, age); acemetacin (n=4, age); haloperidol (n=3, age); domperidone (n=2, age); glimepiride (n=2, age); amitriptyline (n=2, age); fesoterodine (n=2, age); ketotifen (n=2, age); mirabegron (n=2, age); quinolone (n=2, age); theophylline (n=2, age); carbamazepine (n=2, age); paroxetine (n=2, age); oxybutynin (n=2, age); aripiprazole (n=2, age); risperidone (n=2, duration); prasugrel (n=1, age); primidone (n=1, age); clonazepam (n=1, age); tolterodine (n=2, age); doxylamine (n=1, age); flavoxate (n=1, age); lorazepam (n=1, age); opipramol (n=1, age); pioglitazone (n=1, age); levofloxacin (n=1, age); silodosin (n=1, age); propafenone (n=1, age); alprazolam (n=1, age); codeine (n=1, age); sulpiride (n=1, age); propranolol (n=1, age); sennosides (n=1, age); hydroxyzine (n=1, age); naftidrofuryl (n=1, age).

### STOPP v3

PIM-A (age/dose/duration-dependent), n = 14 unique items:

PPI (n=92; age n=3, duration n=89); gliclazide (n=22, age); NSAID > 3 months (n=9, duration); tramadol (n=7, age); atorvastatin (n=5, age); rosuvastatin (n=2, age); glimepiride (n=2, age); ketotifen (n=2, age); nebivolol (n=1, age); mirtazapine (n=1, age); fentanyl (n=1, age); hydroxyzine (n=1, age); ASA > 100 mg (n=1, dose); benzodiazepine > 4 weeks (n=1, duration).

PIM-C (clinical condition/laboratory/event- or interaction-dependent), grouped by triggering domain (Table S3):

**Table S3. STOPP v3 PIM-C items grouped by triggering clinical domain (N = 397).**

| Clinical domain/trigger | Total n | All items (drug - condition, n) |
| --- | --- | --- |
| Dementia | 42 | quetiapine (n=22); Ginkgo biloba (n=6); piracetam (n=4); olanzapine (n=3); trospium (n=3); aripiprazole (n=1); fesoterodine (n=1); propiverine (n=1); tolterodine (n=1) |
| Urinary incontinence / BPH | 22 | furosemide (n=20); alverine (n=1); duloxetine (n=1) |
| Drug-drug interaction | 20 | ASA + clopidogrel (n=5); NSAID + ASA (n=2); NSAID + NSAID (n=1); PPI + PPI (n=1); donepezil + bisoprolol (n=1); donepezil + propranolol (n=1); haloperidol + quetiapine (n=1); simethicone + otilonium bromide (n=1); rivaroxaban + ASA (n=1); rivaroxaban + clopidogrel (n=1); rivaroxaban + carvedilol (n=1); amiodarone + apixaban (n=1); amiodarone + rivaroxaban (n=1); dabigatran + ranolazine (n=1); edoxaban + carvedilol (n=1) |
| Low GFR (renal impairment) | 17 | NSAID (n=9); ASA (n=5); apixaban (n=1); metformin (n=1); spironolactone (n=1) |
| History of falls | 14 | quetiapine (n=2); benzodiazepine, unspecified (n=1); carbamazepine (n=1); citalopram (n=1); doxazosin (n=1); duloxetine (n=1); escitalopram (n=1); mirtazapine (n=1); olanzapine (n=1); paroxetine (n=1); sertraline (n=1); tramadol (n=1); trospium (n=1) |
| Heart failure / low EF | 3 | digoxin in HFpEF (n=3) |
| History of delirium | 2 | quetiapine (n=2) |
| Hyperkalemia | 1 | perindopril (n=1) |
| History of stroke | 1 | sulpiride (n=1) |

## Non-Adjudicable Condition-Dependent Items

Condition-dependent (PIM-C) items and duration-dependent items were applied only when the qualifying information (indication, duration of use, comorbidity, laboratory value, frailty or life expectancy) was documented in the electronic health record. Because these clinical conditions were not captured as a discrete structured variable, patients in whom the information was not documented cannot be separated retrospectively from patients in whom the condition was genuinely absent, and a sensitivity analysis restricted to fully adjudicable patients is therefore not possible. To make the maximum possible extent of under-ascertainment transparent, Table S4 reports, for the most consequential items, the number of patients receiving the index drug, the number in whom the item was coded as a PIM, and the difference. The difference comprises both patients in whom the criterion was genuinely not met and patients in whom the qualifying information was missing; it therefore represents an upper bound of possible under-ascertainment for that item. For drugs listed in PRISCUS 2.0 with an age-only criterion (e.g., NSAIDs, PPIs), the PRISCUS count captures all users aged 65 years or older and serves as the denominator. Because PRISCUS 2.0 is almost entirely age-based, it is essentially unaffected by this limitation; the potential under-ascertainment concerns the Beers 2019 and STOPP v3 lists.

**Table S4. Upper bound of non-adjudicable condition-dependent items for the most consequential criteria (N = 397).**

| **Criterion (list)** | **Index drug(s)** | **Patients receiving index drug, n** | **Coded as PIM, n** | **Not coded (criterion not met or information not documented), n** | **Information required for adjudication** |
| --- | --- | --- | --- | --- | --- |
| ASA for primary cardiovascular prevention (Beers, STOPP) | acetylsalicylic acid | 104 | 0 | 104 | Indication (primary vs secondary prevention) |
| NSAID > 3 months or without gastroprotection (STOPP) | NSAIDs | 20 | 9 | 11 | Duration of use |
| PPI > 8 weeks without indication (Beers, STOPP, PRISCUS) | PPIs | 93 | 92 (STOPP; 89 duration + 3 age), 92 (Beers) | 1 to 4 | Duration of use, indication |
| Benzodiazepine or Z-drug > 4 weeks (STOPP) | benzodiazepines, Z-drugs | 3 | 1 | 2 | Duration of use |
| Statin in limited life expectancy or frailty (STOPP) | statins | 64 | 0 | 64 | Life expectancy, frailty status |
| Antipsychotic in dementia (Beers, STOPP) | quetiapine | 40 | 36 (Beers), 22 (STOPP) | 4 (Beers), 18 (STOPP) | Dementia diagnosis |
| SSRI with history of falls (Beers) | escitalopram, citalopram, sertraline | 50 | 22 | 28 | Documented fall history |

ASA, acetylsalicylic acid; NSAID, non-steroidal anti-inflammatory drug; PPI, proton pump inhibitor; SSRI, selective serotonin reuptake inhibitor; BZD, benzodiazepine; Z-drugs, zolpidem, zopiclone, zaleplon. The 'not coded' column cannot distinguish undocumented from genuinely absent conditions and is an upper bound.

## PIM-ALL: Combination Rule and Cohort Summary

For each patient, PIM-ALL was derived by pooling every medication coded as PIM-A or PIM-C under PRISCUS 2.0, Beers 2019, or STOPP v3, and removing duplicate entries so that a medication independently flagged by more than one list (e.g., PPI overuse, flagged by all three lists, or quetiapine in dementia, flagged by both Beers 2019 and STOPP v3) contributed only once to the patient’s total PIM-ALL count. This is consistent with the definition of PIM-ALL as a comprehensive, non-redundant summary measure rather than a simple sum of the three list-specific counts.

The distribution of the number of PIM-ALL items per patient is shown in Table S5. Of the 397 patients, 233 (58.7%) had at least one PIM and 60 (15.1%) had three or more; the maximum was 7.

**Table S5. Distribution of the number of PIM-ALL items per patient (N = 397).**

| **Number of PIM-ALL items** | **n** | **%** |
| --- | --- | --- |
| 0 | 164 | 41.3 |
| 1 | 103 | 25.9 |
| 2 | 70 | 17.6 |
| 3 | 27 | 6.8 |
| 4 | 18 | 4.5 |
| 5 | 7 | 1.8 |
| 6 | 7 | 1.8 |
| 7 | 1 | 0.3 |
| At least 1 PIM (total) | 233 | 58.7 |
